# Supplementary material for: Human risk to tick encounters in the southeastern United States estimated with spatial distribution modeling
Source: PLoS Negl Trop Dis. 2024 Feb 14;18(2):e0011919. doi: 10.1371/journal.pntd.0011919 (PMC10898775; doi:10.1371/journal.pntd.0011919)
Supplement: S1 Table — (DOCX) [file pntd.0011919.s001.docx]

**S1 Table .** Correlation values for environmental variables that contributed 10% or more to each environmental niche model.

| ***Amblyomma americanum* model** | | | | | |
| --- | --- | --- | --- | --- | --- |
| **Variable** | **Elevation** | **Minimum temperature** | **Maximum temperature** | **Vapor pressure** |  |
| **Elevation** | 1.00000 | -0.70238 | -0.72356 | -0.69379 |  |
| **Minimum temperature** | -0.70238 | 1.00000 | 0.96511 | 0.99586 |  |
| **Maximum temperature** | -0.72356 | 0.96511 | 1.00000 | 0.94482 |  |
| **Vapor pressure** | -0.69379 | 0.99586 | 0.94482 | 1.00000 |  |
| ***Dermacentor variabilis* model** | | | | | |
| **Variable** | **Elevation** | **Vapor pressure** | **Precipitation** | **Maximum temperature** | **Dead belowground biomass** |
| **Elevation** | 1.00000 | -0.69379 | -0.2663 | -0.72356 | 0.73555 |
| **Vapor pressure** | -0.69379 | 1.00000 | 0.4377 | 0.94482 | -0.46325 |
| **Precipitation** | -0.2663 | 0.4377 | 1.00000 | 0.37634 | -0.14292 |
| **Maximum temperature** | -0.72356 | 0.94482 | 0.37634 | 1.00000 | -0.51705 |
| **Dead belowground biomass** | 0.73555 | -0.46325 | -0.14292 | -0.51705 | 1.00000 |
| ***Ixodes scapularis* model** | | | | | |
| **Variable** | **Maximum temperature** | **Evapotranspiration** | **Gross primary productivity** | **Vegetation indices** | **Soil organic matter** |
| **Maximum temperature** | 1.00000 | -0.00233 | 0.11045 | 0.17802 | -0.43516 |
| **Evapotranspiration** | -0.00233 | 1.00000 | 0.57713 | 0.44479 | 0.01016 |
| **Gross primary productivity** | 0.11045 | 0.57713 | 1.00000 | 0.92886 | -0.01242 |
| **Vegetation indices** | 0.17802 | 0.44479 | 0.92886 | 1.00000 | -0.0152 |
| **Soil organic matter** | -0.43516 | 0.01016 | -0.01242 | -0.0152 | 1.00000 |
