# Supplementary material for: Human risk to tick encounters in the southeastern United States estimated with spatial distribution modeling
Source: PLoS Negl Trop Dis. 2024 Feb 14;18(2):e0011919. doi: 10.1371/journal.pntd.0011919 (PMC10898775; doi:10.1371/journal.pntd.0011919)
Supplement: S2 Table — (DOCX) [file pntd.0011919.s002.docx]

| **S2 Table .** Lambda file results for environmental variables in each Maxent environmental niche model. | | | | | | | |
| --- | --- | --- | --- | --- | --- | --- | --- |
| **Feature type** | ***Amblyomma americanum*** | | | ***Dermacentor variabilis*** | | | ***Ixodes scapularis*** |
|  | **Model 1** | **Model 2** | **Model 3** | **Model 1** | **Model 2** | **Model 3** | **Model 1** |
| **Raw features** | Living  aboveground biomass | Living  aboveground biomass | Living  aboveground biomass | Living  aboveground biomass | Living  aboveground biomass | Living  aboveground biomass | Living aboveground biomass |
|  | Living belowground biomass | Living belowground biomass | Living belowground biomass | Living belowground biomass | Living belowground biomass | Living belowground biomass | Living belowground biomass |
|  | Burned area | Burned area | Burned area | Burned area | Burned area | Burned area | Burned area |
|  | Litter | Litter | Litter | Litter | Litter | Litter | Litter |
|  | Soil organic matter | Soil organic matter | Soil organic matter | Soil organic matter | Soil organic matter | Soil organic matter | Soil organic matter |
|  | Dead aboveground biomass | Dead aboveground biomass | Dead aboveground biomass | Dead aboveground biomass | Dead aboveground biomass | Dead aboveground biomass | Dead aboveground biomass |
|  | Dead belowground biomass | Dead belowground biomass | Dead belowground biomass | Dead belowground biomass | Dead belowground biomass | Dead belowground biomass | Dead belowground biomass |
|  | Elevation | Elevation | Elevation | Elevation | Elevation | Elevation | Elevation |
|  | Evapotranspiration | Evapotranspiration | Evapotranspiration | Evapotranspiration | Evapotranspiration | Evapotranspiration | Evapotranspiration |
|  | Gross primary productivity | Gross primary productivity | Gross primary productivity | Gross primary productivity | Gross primary productivity | Gross primary productivity | Gross primary productivity |
|  | Leaf area index | Leaf area index | Leaf area index | Leaf area index | Leaf area index | Leaf area index | Leaf area index |
|  | Land surface temperature | Land surface temperature | Land surface temperature | Land surface temperature | Land surface temperature | Land surface temperature | Land surface temperature |
|  | Net primary productivity | Net primary productivity | Net primary productivity | Net primary productivity | Net primary productivity | Net primary productivity | Net primary productivity |
|  | Precipitation | Precipitation | Precipitation | Precipitation | Precipitation | Precipitation | Precipitation |
|  | Maximum temperature | Maximum temperature | Maximum temperature | Maximum temperature | Maximum temperature | Maximum temperature | Maximum temperature |
|  | Minimum temperature | Minimum temperature | Minimum temperature | Minimum temperature | Minimum temperature | Minimum temperature | Minimum temperature |
|  | Vapor pressure | Vapor pressure | Vapor pressure | Vapor pressure | Vapor pressure | Vapor pressure | Vapor pressure |
|  | Vegetation indices | Vegetation indices | Vegetation indices | Vegetation indices | Vegetation indices | Vegetation indices | Vegetation indices |
| **Quadratic features** | Soil organic matter | Soil organic matter | Soil organic matter | Living belowground biomass | Living belowground biomass | Living belowground biomass | Litter |
|  | Evapotranspiration | Land surface temperature | Land surface temperature | Elevation | Elevation | Elevation | Elevation |
|  | Land surface temperature |  |  | Evapotranspiration | Maximum temperature | Evapotranspiration |  |
|  |  |  |  | Maximum temperature |  | Land surface temperature |  |
|  |  |  |  |  |  | Maximum temperature |  |
| **Product features** | Living belowground biomass*  Precipitation | Living belowground biomass*  Net primary productivity | Living belowground biomass*  Elevation |  |  |  | Litter*Dead belowground biomass |
|  | Living belowground biomass*Net primary productivity | Living belowground biomass*  Precipitation | Living belowground biomass*  Net primary productivity |  |  |  | Soil organic matter*Leaf area index |
|  | Litter*  Precipitation | Litter*  Precipitation | Living belowground biomass*  Precipitation |  |  |  | Soil organic matter*Maximum temperature |
|  | Soil organic matter*  Precipitation | Litter*Vapor pressure | Litter*  Minimum temperature |  |  |  | Soil organic matter*Vegetation indices |
|  | Elevation*  Evapotranspiration | Soil organic matter*  Evapotranspiration | Litter*  Vapor pressure |  |  |  | Evapotranspiration*Gross primary productivity |
|  | elevation*Minimum temperature | Soil organic matter*Precipitation | Soil organic matter*  Evapotranspiration |  |  |  | Leaf area index*Maximum temperature |
|  | Evapotranspiration*  Minimum temperature | Dead belowground biomass*  Vegetation indices | Soil organic matter*  Precipitation |  |  |  | Net primary productivity*  Vegetation indices |
|  | Gross primary productivity*Net primary productivity | Elevation*  Evapotranspiration | Dead belowground biomass*  Leaf area index |  |  |  |  |
|  | Leaf area index*  Maximum temperature | Elevation*  Minimum temperature | elevation*  Evapotranspiration |  |  |  |  |
|  | Net primary productivity*  Minimum temperature | Evapotranspiration*  Precipitation | elevation*  Minimum temperature |  |  |  |  |
|  | Precipitation*  Minimum temperature | Net primary productivity*  Minimum temperature | Evapotranspiration*  Minimum temperature |  |  |  |  |
|  |  | Precipitation*  Minimum temperature | Evapotranspiration*  Vapor pressure |  |  |  |  |
|  |  |  | Net primary productivity*  Vegetation indices |  |  |  |  |
|  |  |  | Precipitation*  Minimum temperature |  |  |  |  |
| **Forward hinge features** | Minimum temperature | Land surface temperature | Maximum temperature | Soil organic matter | Evapotranspiration | Precipitation |  |
|  | Maximum temperature | Maximum temperature | Evapotranspiration | Precipitation | Minimum temperature | Soil organic matter |  |
|  | Soil organic matter | Evapotranspiration | Minimum temperature | Minimum temperature | Precipitation | Minimum temperature |  |
|  | Evapotranspiration | Minimum temperature | Soil organic matter |  |  |  |  |
|  |  |  | Litter |  |  |  |  |
| **Reverse hinge features** | Net primary productivity | Elevation | Net primary productivity | Evapotranspiration | Elevation | Dead belowground biomass |  |
|  | Minimum temperature | Evapotranspiration | Gross primary productivity | Vegetation indices | Dead belowground biomass | Evapotranspiration |  |
|  | Vapor pressure | Minimum temperature | Precipitation | Dead belowground biomass | Evapotranspiration | Living belowground biomass |  |
|  | Evapotranspiration | Living belowground biomass | Evapotranspiration | Living belowground biomass | Minimum temperature | Soil organic matter |  |
|  | Elevation | Vapor pressure | Living belowground biomass | Dead belowground biomass | Vapor pressure | Vapor pressure |  |
|  | Litter | Litter | Dead belowground biomass | Vapor pressure | Living belowground biomass | Minimum temperature |  |
|  | Elevation | Net primary productivity | Elevation | Net primary productivity | Land surface temperature | Net primary productivity |  |
|  | Living belowground biomass | Gross primary productivity | Maximum temperature | Elevation | Dead belowground biomass | Precipitation |  |
|  | Vapor pressure | Dead belowground biomass | Litter | Minimum temperature | Net primary productivity | Elevation |  |
|  | Maximum temperature | Maximum temperature | Land surface temperature |  |  | Gross primary productivity |  |
|  | Evapotranspiration | Leaf area index | Vapor pressure |  |  | Maximum temperature |  |
|  | Dead aboveground biomass | Precipitation | Leaf area index |  |  |  |  |
|  | Precipitation |  | Burned area |  |  |  |  |
|  | Leaf area index |  | Living aboveground biomass |  |  |  |  |
|  | Burned area |  | Minimum temperature |  |  |  |  |
|  | Dead belowground biomass |  |  |  |  |  |  |
|  | Land surface temperature |  |  |  |  |  |  |
|  | Gross primary productivity |  |  |  |  |  |  |
| **Threshold features** | Landcover | Landcover | Landcover | Landcover | Landcover | Landcover |  |
|  | Hydrologic soil group | Hydrologic soil group | Hydrologic soil group | Hydrologic soil group | Hydrologic soil group | Hydrologic soil group |  |
