# Supplementary material for: Human risk to tick encounters in the southeastern United States estimated with spatial distribution modeling
Source: PLoS Negl Trop Dis. 2024 Feb 14;18(2):e0011919. doi: 10.1371/journal.pntd.0011919 (PMC10898775; doi:10.1371/journal.pntd.0011919)
Supplement: Attachment — Submitted filename: Response2Reviewers_final_ENM.docx [file pntd.0011919.s003.docx]

Reviewers felt that there was considerable value to this study but also were clear that many aspects of this submission need to be improved.  The authors are encouraged to carefully consider the comments of the reviewers and ensure that they are adequately addressed in a revision.  Also, please be sure to carefully review the submission for spelling and gramma. This is important as PLoS does not copy-edit manuscripts.

Reviewer's Responses to Questions

Key Review Criteria Required for Acceptance
As you describe the new analyses required for acceptance, please consider the following:

Methods
-Are the objectives of the study clearly articulated with a clear testable hypothesis stated?
-Is the study design appropriate to address the stated objectives?
-Is the population clearly described and appropriate for the hypothesis being tested?
-Is the sample size sufficient to ensure adequate power to address the hypothesis being tested?
-Were correct statistical analysis used to support conclusions?
-Are there concerns about ethical or regulatory requirements being met?

Reviewer #1: -Are the objectives of the study clearly articulated with a clear testable hypothesis stated? Yes
-Is the study design appropriate to address the stated objectives? yes
-Is the population clearly described and appropriate for the hypothesis being tested? Yes
-Is the sample size sufficient to ensure adequate power to address the hypothesis being tested? Yes
-Were correct statistical analysis used to support conclusions? See comments
-Are there concerns about ethical or regulatory requirements being met? No

Reviewer #2: The methods are clearly stated. It is less clear if there are sufficient data for this analysis. The data were collected over 8 years across a wide area. It is unclear if the crews worked equally across the region in all years and all areas. The inter annual variation in ticks could cause the models to provide errant results if the trends are artifacts of an interplay between geography and year.

Agree, clarification added

There is also little justification for the choice of MaxEnt as the only tool used. There are many other options that could be used for this type of analysis.

Agreed, clarified as suggested

Reviewer #3: Lines 105, 305 (and others). The authors are conflating the presence of the tick with the occurrence of a reported human-tick encounter.

agreed, changed to human-tick encounter

Line 124. Why was a binary logit model (tick species present/absent) used for this analysis? Models or tests like a chi-squared test of abundance by season would utilize more of the data and variation temporally. As mentioned below, models/tests should be broken down by life stage.

Our data are opportunistic samples, thus they are more adequate for modeling potential suitability (presence/absence) than abundance. On the second point, we agree: models were broken down by tick life stage, but we kept the MaxEnt models for the species (not life stage) to maximize the sample sizes (presences) used to train and test our models. Future research and collections that increase sample size by life stage could allow for life stage specific models.

Line 126. Passive surveillance does not give “true” phenology, but rather the seasonal variation in host-seeking tick-human encounters. This would be modified by things like climatic conditions affecting tick behavior as well as human behavior in tick habitats.

Agreed, added clarification

Line 132. Given the small size of larval and nymphal ticks, how confident are the authors that the submitted ticks did not originate from a different FIA plot (>1.6 km from the GPS point of the reported plot)?

We are quite confident that ticks were not picked up from other plots because they could only visit one plot a day given the amount of work they had to do at each site. To clarify this and reduce confusion we added more detail here.

Lines 136-138. Given the large variation in numbers of tick species by life stage, the fit ENM may predict highly suitable habitat in places where larvae were collected, just because of their higher numbers relative to other life stages. Were the ENM fit with present/absent or count (# ticks) data?

This is a great question. Maxent uses presence only data instead of abundance. Thus, our models do not link suitability to abundance (i.e., higher abundance is not linked to higher suitability). The bias in sampling is problematic when a species’ range is extensive, and the sampling is spatially clustered or localized. Our sampling spanned the majority of the species’ range. We acknowledged in the manuscript that one shortcoming of our study is the sample imbalance by life stage. This can only be addressed by increasing the survey efforts, by season. .

Line 140. Were the gridded environmental data originally or projected to be on an equal-area projection for fitting the ENMs? Use of non-projected data could violate the equal-area assumption of ENMs since such a large area of land was used in the modeling.

We used the NAD 1983 geographic coordinate system. Internally, Maxent matches the location of presence data (latitude and longitude coordinates) with environmental variables at those locations. Model fitting is then carried out in environmental space; thus, an equal-area projection is not necessary for model fitting.

Line 143. Why were averaged rasters across the collection dates used instead of the individual rasters per unique collection? Averaging can obscure the variation in environmental conditions under which a tick was found on a forestry worker.

Creating an average environmental raster by date provides a method for assigning an environmental variable value to each data point. Because our tick sampling spanned eight years and many dates associated with those collections, there are many environmental variables, for each narrow time period (16 days or monthly). It is not possible to run models with temporal replicates (e.g., monthly or yearly) of a particular variable (e.g., vegetation index). We opted for the aggregate average for environmental variables and not extreme weather events.

Line 151. Did you assess auto-correlation between the presence sites?

We did not assess auto-correlation of presence sites, but this aspect is relevant to Maxent modeling in the context of environmental sampling and correlation. We added information in the manuscript regarding the algorithm’s capacity to handle correlation among predictor variables. Correlations for variables that contributed 10% or more to each model can now be found in Supplement Table 1.

Line 152. How were pseudo-absence points selected when fitting the models or were background points used? How many points were used?

This information was missing from our methods. We have added it in the revised manuscript. We used 10,000 random background samples to fit each species’ model.

Line 157. While Maxent is a generally more stable framework in the face of correlated variables than other frameworks (like regression), it is not completely immune to difficulties arising from correlation of covariates (Sillero & Barbosa 2020, <https://www.tandfonline.com/doi/full/10.1080/13658816.2020.1798968>). How much correlation was present between the covariates included in the model?

We agree with this statement and clarified this in the text and added into Supplement Table 1.

Line 191. Why were dry ice traps used for the active surveillance model validations? Dry ice traps are not very effective at collecting Ixodes and Dermacentor ticks and efficacy varies by life stage (e.g., Schulze et al. 1997, doi: 10.1093/jmedent/34.6.615; Falco & Fish 1992, doi: 10.1007/BF01219108). Also, were these traps just run for a single night per month? Given factors like variation in attractiveness of the trap and climatic impacts on tick behavior, lack of collection does not necessarily mean lack of tick presence in these locations.

Agreed, clarification added. We attempted to drag the sites as well but did not complete this at all sites each time period, so it was excluded from the study.

Lines 172-176. Targeted surveillance only occurred at three sites, and all were negative for the tick species of interest. This seems like too small of a sample size for model validation and only assessed the model’s ability to predict “not suitable” habitat types. Validation of predictions of “suitable” from targeted surveillance seem lacking here.

We used sites where ticks were found on foresters (258 sites for *A. americanum*, 90 sites for *D. variabilis*, and 36 sites for *I. scapularis*) for model validation. Although the more sites used to train each Maxent model the better, this approach only requires 15 presences to build models for predicted species distributions. For all of our models we had an AUC of 0.8 or above and we used 10% training threshold omission error to ensure the best model predictions. This is stated in the manuscript methods. Additionally, we also investigated the use of CDC datasets for a spatially coarser evaluation of the models.

Line 200. The CDC dataset may not represent the true distribution of presence for each species since tick surveillance is not standardized across the United States. Lack of reported presence in the CDC dataset may represent a lack of surveillance conducted in the county and not necessarily indicate that the tick species is not present in the county. Thus, leading to incorrect estimation of model commission error.

Agreed, but it is currently our best method at the moment. We did address this in the manuscript and attempted to clarify it in writing.

--------------------

Results
-Does the analysis presented match the analysis plan?
-Are the results clearly and completely presented?
-Are the figures (Tables, Images) of sufficient quality for clarity?

Reviewer #1: -Does the analysis presented match the analysis plan? Yes
-Are the results clearly and completely presented? See comments
-Are the figures (Tables, Images) of sufficient quality for clarity? yes

Reviewer #2: The results are clear and make sense. The numbers of ticks collected is just so small spread across this geographic region that is makes the models less useful. The figures are a bit difficult to discern in black and white.

While larger training sample sizes may increase the performance of ENMs, Maxent tends to generate reliable models with low sample sizes. For all of our models we had an AUC of 0.8 or above and we used 10% training threshold omission error to ensure the best model predictions. This is stated in the manuscript methods.

We opted for black and white maps for color blind individuals and printed versions of the manuscript.

Reviewer #3: The analysis presented does match the analysis plan presented. However, these will need to be updated based on amendments made to the modeling outlines in the Methods.
Table 3. What shape of relationships (linear, quadratic, etc) did these variables have in the models? The methods indicated different forms were trialed across the different models and species.

We added results from the lambda file for all environmental niche models into Supplement table 2.

--------------------

Conclusions
-Are the conclusions supported by the data presented?
-Are the limitations of analysis clearly described?
-Do the authors discuss how these data can be helpful to advance our understanding of the topic under study?
-Is public health relevance addressed?

Reviewer #1: -Are the conclusions supported by the data presented? see comments, but in general yes
-Are the limitations of analysis clearly described? yes
-Do the authors discuss how these data can be helpful to advance our understanding of the topic under study? yes
-Is public health relevance addressed?yes

Reviewer #2: It isn't clear what results add to the understanding of tick populations given the uncertainty of the model data.

This study was done to create predicted distributions of human-tick encounters which can be used by forestry personnel to help protect themselves from tick bites by reinforcing areas where personal protective equipment should be worn. This was explained in the conclusion.

Reviewer #3: The conclusions are generally supported by the data presented, but the authors’ language conflates the presence of the tick with the occurrence of human-tick encounters. Data from passive tick submissions provide information on human-tick encounters and not necessarily of the presence of the tick itself given difference in the host-seeking and host preference of each tick species and even life stage. Also, the authors do not make distinctions between temporal variation in tick species presence by life stage, but rather lump all life stages together for each tick species. Abundance and host preference can vary across life stages such that the risk of tick-borne disease can vary across time.
Agreed, added “human-tick encounter” throughout manuscript and modeled tick activity for season by life stage.
--------------------

Editorial and Data Presentation Modifications?
Use  this section for editorial suggestions as well as relatively minor modifications of existing data that would enhance clarity. If the only modifications needed are minor and/or editorial, you may wish to recommend “Minor Revision” or “Accept”.

Reviewer #1: (No Response)

Reviewer #2: (No Response)

Reviewer #3: Lines 57-58. These tick-borne diseases (and syndrome) do not need to be capitalized.

Agreed, edited as suggested

Lines 87, 89. Parentheses and brackets are mixed together for references.

Agreed, edited as suggested

Line 92. The phrase “of host” at the end of the sentence is confusing. Please clarify if you are referring to the tick species or the host species for the ticks.

Agreed, edited as suggested

Maps in figures would benefit from using projected coordinate systems (equal-area projections).

We opted to not use an equal-area projection because it was distorting (making it hard to discern between) the ecoregions and numerical values on the map.

--------------------

Summary and General Comments
Use  this section to provide overall comments, discuss strengths/weaknesses of the study, novelty, significance, general execution and scholarship. You may also include additional comments for the author, including concerns about dual publication, research ethics, or publication ethics. If requesting major revision, please articulate the new experiments that are needed.

Reviewer #1: Major Comments

The use of season as four variables seems very broad as summer encompasses June 21-September 21, in which temperature and life stages of ticks can vary a lot. I wonder if the authors tried to use month rather than season, especially given that the data given in figure 1 is by month not season. There is also the possibility of using week of year. Using AIC could help determine which of those predictor variables are the best, as it is pretty much a given that month would be significant for ticks. But is it biologically significant. We know that spring, summer and fall in general are more likely for ticks to be out, but for human risk, having a more narrower definition of what time of year.

We used season because this is based on day length and is known to be associated with tick presence throughout scientific literature and was clarified in the manuscript.

I’m concerned about not using the actual coordinates of the encounter site. Event those the exact locations are kept confidential, as an author works for the USDA FIA and there are no coordinates posted in this MS,

This is why we coarsened the spatial resolution of the pixel for each individual raster. Added “ , for ticks collected while walking to and from plots and GPS recorded outside property boundaries,”. The FIA uses this approach to keep sites confidential (we do not know the real sites) but all sites are real and ‘swapped’ with nearly equivalent sites in terms of environmental variables.

Can you better explain why you did not assess the raster images for autocorrelation and what was the method for Maxent to discern. Also what does it mean that the land cover variables were not balanced for I. scapularis.  Do you have a rationale for why this is acceptable?

Agreed, clarification added

Why was the overnight monthly trapping with dry ice only from March to June?

Most ticks are collected during this period in the area. We clarified as suggested

What exactly are the sensitivity tests used? There are a wide range of test that are labeled as “Sensitivity” and please explain why you are performing these test.

Agreed, clarified as suggested

In the discussion section for Ixodes scapularis, can you discuss the inability to include landcover in you ENM model.

Agreed, clarified as suggested

Minor Comments
Line 66-68: The sentence “Environmental niche models…” is unclear. These models do not estimate distribution maps, but create maps with estimated distributions.

Agreed, edited as suggested

Line 71- Remove “potential”

Agreed, edited as suggested

Line  71-73: You use “parts of” twice, which is redundant, You could probably skip the California bit as the sentences primary talks about countries or emphasized the western US instead of just California.

Agreed, edited as suggested

Line 84: I don’t think you should use a numbered citation in the beginning of the sentence. Put the authors name and place the number where the year would be or rewrite the sentence so that the citation is at the end. Maybe, “Specifically,  prescribed burns can reduce tick abundance by factors such as heat exposure or decrease in soil moisture [26]”

Agreed, edited as suggested

Line 86: add  “also” between “and precipitation) have” and  “been associated with”

Agreed, edited as suggested

Line 99-100: The sentence “In other words… “ is not needed.

Agreed, edited as suggested

Line 145-146:  I’m not entirely sure what you mean by “compensating for potential spatial sampling errors…” and why you would increase from 500 meters to 4.8 km as opposed to 1.6km.

Agreed, clarified as suggested

Lines 269-271: I’m curious if the opposite was true, did you place traps in areas that the model predicted as suitable so as to confirm the model correctly predicted sites where the ticks were found?

Traps were not placed in areas that the models predicted were suitable but because GPS presence records for where human-tick encounters were located were used to train the algorithm we expect that models would already be associated with tick presences.

Line 275-278: What exactly are these sensitivity values? Are they percentages? What do they tell the reader?

Agreed, clarified as suggested

Line 300: You have abiotic twice.

Removed the word repetition.

Line 319: You give five variables

Agreed, edited as suggested

Line 350: Foresters frequently encountered…

Agreed, edited as suggested

Line 351-354: Six environmental variables contributed to the environmental niche models with   consistent variables including elevation and landcover; and the remaining four variables (maximum temperature, precipitation, vapor pressure, and dead belowground biomass)  varied in contribution for each model.

Agreed, edited as suggested

Line 355-357: I’m not completely sure “but” is appropriate in this sentence. Maybe “and”

Agreed, edited as suggested

Line 363-371: Check the tenses in this sentence. There are many instances of using were where was should be used.

Agreed, edited as suggested

Line 378-379: Can you what increasing dead belowground biomass would do and how it will affect ticks?
Agreed, clarified as suggested

Line 408: What are these variable that are associated with questing and surviving.

Agreed, clarified as suggested

Reviewer #2: Overall, the paper looks at an important concept of understanding tick risk in space. It is unclear if the data are sufficient to support useful findings across the southeastern region.

Our model results showed that these niche models did a good job at predicting tick presence across Southeast, this is explained above.

Reviewer #3: Butler et al. used passive submissions of three tick species from forestry workers in the southeastern United States to estimate the ecological niche of each species and determine spatial and temporal risk of tick encounters. This is an important study to use passive surveillance data to inform risk of human-tick encounters and an interesting population (forestry workers) to use given their unique exposure to tick habitats. I do have some concerns on how the data were used in the analysis and conclusions drawn from these results. Below are my largest concerns.

The authors did not differentiate between tick life stages in the data analysis. The timing of host-seeking varies by life stage for tick species so grouping all life stages together for a tick species obscures the variation in human encounter rates with different life stages. Also, the host preference and density of each tick species and life stage varies, thus skewing monthly percent encounters to more abundant life stages that bite humans. Additionally, the risk of tick-borne disease transmission can vary across life stages of a species (e.g., larval Ixodes scapularis do not transmit Borrelia burgdorferi since this bacterium is not transovarially transmitted), so assessment of risk from the human-tick encounters can vary across the year. Differentiation should be made between life stages for tick species in the analysis, especially the GLM for seasonal differences.

Agreed, edited as suggested

Throughout, the authors’ language conflates the presence of the tick with the occurrence of human-tick encounters. Data from passive tick submissions provide information on human-tick encounters and not necessarily of the presence of the tick itself given the host-seeking and host preference of each tick species and even life stage.

Agreed, changed to human-tick encounter

More details should be provided for the ecological niche modeling. It appears that correlation between covariates were not taken into account and environmental covariates may have been calculated from unprojected coordinate systems. A list of some common mistakes made in ecological niche modeling (and explanation of how these violate assumptions of the modeling framework) can be found here: Sillero & Barbosa 2020, <https://www.tandfonline.com/doi/full/10.1080/13658816.2020.1798968>

Agreed, clarification added

--------------------

PLOS authors have the option to publish the peer review history of their article (<a href="<https://journals.plos.org/plosntds/s/editorial-and-peer-review-process#loc-peer-review-history>" target="_blank">what does this mean?</a>). If published, this will include your full peer review and any attached files.

If you choose “no”, your identity will remain anonymous but your review may still be made public.

<b>Do you want your identity to be public for this peer review?</b> For information about this choice, including consent withdrawal, please see our <a href="<https://www.plos.org/privacy-policy>" target="_blank">Privacy Policy</a>.

Reviewer #1: Yes: Samniqueka Halsey

Reviewer #2: No

Reviewer #3: No
