# Supplementary material for: Human risk to tick encounters in the southeastern United States estimated with spatial distribution modeling
Source: PLoS Negl Trop Dis. 2024 Feb 14;18(2):e0011919. doi: 10.1371/journal.pntd.0011919 (PMC10898775; doi:10.1371/journal.pntd.0011919)
Supplement: Attachment — Submitted filename: Nov30-Response.docx [file pntd.0011919.s004.docx]

Manuscript ID: PNTD-D-23-00705R2

1) Please ensure that the CRediT author contributions listed for every co-author are completed accurately and in full. At this stage, "All Authors" require contributions. Please ensure that the full contributions of each author are acknowledged in the ""Add/Edit/Remove Authors"" section of our submission form.

# **R. A. Butler**

- Roles: Data Curation, Formal Analysis, Methodology, Validation, Visualization, Writing Original Draft, Writing Review and Editing
- Email: rbutle25@vols.utk.edu
- Affiliation: Department of Entomology and Plant Pathology, University of Tennessee, Knoxville, Tennessee, USA
- Orcid ID:  <https://orcid.org/0000-0003-1865-9746>

# **M. Papeş**

- Roles: Formal Analysis, Methodology, Supervision, Visualization, Writing Review and Editing
- Email: mpapes@utk.edu
- Affiliation: Department of Ecology & Evolutionary Biology, University of Tennessee, Knoxville, Tennessee, USA
- Orcid ID: <https://orcid.org/0009-0007-9662-5580>

# **J. T. Vogt**

- Roles: Funding Acquisition, Methodology, Project Administration, Resources, Supervision, Writing Review and Editing
- Email: [james.t.vogt@usda.gov](mailto:james.t.vogt@usda.gov)
- Affiliation: United States Department of Agriculture Forest Service, Southern Research Station, Knoxville, Tennessee, USA
- Orcid ID: <https://orcid.org/0000-0002-5734-1784>

# **D. J. Paulsen**

- Roles: Data Curation, Methodology, Writing Review and Editing
- Email: [dpaulsen@utk.edu](mailto:dpaulsen@utk.edu)
- Affiliation: Department of Entomology and Plant Pathology, University of Tennessee, Knoxville, Tennessee, USA
- Orcid ID: no orcid id

# **C. Crowe**

- Roles: Methodology, Validation, Writing Review and Editing
- Email: [christopher.crowe@usda.gov](mailto:christopher.crowe@usda.gov)
- Affiliation: United States Department of Agriculture Forest Service, Southern Research Station, Knoxville, Tennessee, USA
- Orcid ID: no orcid id

# **R. T. Trout Fryxell**

- Roles: Conceptualization, Data Curation, Funding Acquisition, Methodology, Project Administration, Supervision, Validation, Visualization, Writing Review and Editing
- Email: [rfryxell@utk.edu](mailto:rfryxell@utk.edu)
- Affiliation: Department of Entomology and Plant Pathology, University of Tennessee, Knoxville, Tennessee, USA
- Orcid ID: <https://orcid.org/0000-0003-2133-0220>

2)  Please ensure that the funders and grant numbers match between the Financial Disclosure field and the Funding Information tab in your submission form. Note that the funders must be provided in the same order in both places as well. Currently, " The Department of Entomology and Plant Pathology at the University of Tennessee helped support Butler’s graduate stipend and tuition" is missing from the Funding Information.

The USDA Forest Service – Southern Research Station and the Department of Entomology and Plant Pathology at the University of Tennessee were included under funding source.

3) Please amend your detailed Financial Disclosure statement. This is published with the article, therefore should be completed in full sentences and contain the exact wording you wish to be published.

i). State the initials, alongside each funding source, of each author to receive each grant.

ii). State what role the funders took in the study. If the funders had no role in your study, please state: “The funders had no role in study design, data collection and analysis, decision to publish, or preparation of the manuscript.”

iii). If any authors received a salary from any of your funders, please state which authors and which funders.

Edited to include: The Forest Inventory and Analysis Program of the Forest Service, United States Department of Agriculture providing funding for the collection and submission of the ticks used in the study; procured by JTV and RTTF. JTV and CC are employed by the USDA Forest Service Southern Research Station. The Department of Entomology and Plant Pathology at the University of Tennessee helped support RAB’s graduate stipend and tuition.

4) Thank you for stating"Tick encounter data at the county level will be made available on Dryad upon notification of acceptance". Should your submission be accepted, we will require the following information in your Data Availability Statement:

1. The DOI provided by Dryad: The data are located at doi:10.5061/dryad.v41ns1s3n.

5) Some material included in your submission may be copyrighted. According to PLOS’s copyright policy, authors who use figures or other material (e.g., graphics, clipart, maps) from another author or copyright holder must demonstrate or obtain permission to publish this material under the Creative Commons Attribution 4.0 International (CC BY 4.0) License used by PLOS journals. Please closely review the details of PLOS’s copyright requirements here: PLOS Licenses and Copyright. If you need to request permissions from a copyright holder, you may use PLOS's Copyright Content Permission form.

Please respond directly to this email and provide any known details concerning your material's license terms and permissions required for reuse, even if you have not yet obtained copyright permissions or are unsure of your material's copyright compatibility. Once you have responded and addressed all other outstanding technical requirements, you may resubmit your manuscript within Editorial Manager.

Potential Copyright Issues:

[Figure2,3,4,5,6]: Thank you for stating"For figures 2-6; the following link was used as the base layer of the map (<https://www.census.gov/geographies/mapping-files/time-series/geo/carto-boundary-file.html>)".

Please be so kind  and add this direct link to the base layer of the map in the respective figure legends.

**We included that in this in the figure legend.**

Once you have made the necessary changes in Editorial Manager, please re-approve the submission by following the same steps as before.

Please resolve these issues and resubmit your manuscript within 30 days. If you need more time, please email the journal office at plosntds@plos.org. We are happy to grant extensions of up to one month past this due date. If we do not hear from you within 30 days, we will withdraw your manuscript on your behalf.                                                                              

If you have any questions or require assistance, please don't hesitate to get in touch. To help us minimize inbox volumes, we kindly ask that you not reply to this email simply to confirm you have resubmitted your revisions.
